# Measurement properties of core outcomes in patients with tennis elbow

**DOI:** 10.1177/17585732251344264

**Published:** 2025-05-29

**Authors:** Håkon Sveinall, Jens Ivar Brox, Kaia B Engebretsen, Aasne Fenne Hoksrud, Cecilie Røe, Marianne Bakke Johnsen

**Affiliations:** 1 Department of Physical Medicine and Rehabilitation, 155272 Oslo University Hospital, Oslo, Norway; 2 Faculty of Medicine, 60504 Institute for Clinical Medicine, University of Oslo, Oslo, Norway; 3 125798 Norwegian Olympic and Paralympic Committee and Confederation of Sports, Oslo, Norway; 4 Department of Rehabilitation Science and Health Technology, Faculty of Health Sciences, 60499 Oslo Metropolitan University, Oslo, Norway

**Keywords:** Tennis elbow, elbow tendinopathy, validation study, reproducibility of results, minimal clinically important difference, psychometrics, pain measurement, hand strength, muscle strength dynamometer, patient reported outcome measures

## Abstract

**Background:**

The Patient-Rated Tennis Elbow Evaluation (PRTEE), pain on gripping, pain-free, and maximum grip strength are widely used outcomes for tennis elbow. This study tested the measurement properties with a prospective test–retest study design.

**Methods:**

100 participants with tennis elbow were included. The reliability, internal consistency, validity, responsiveness, and the Minimal Important Change (MIC) were evaluated.

**Results:**

The reliability of all measures was acceptable. 8/15 PRTEE items were below the criteria for content validity. For PRTEE total score (0–100), the smallest detectable change 95% (SDC_95%_) was 17 points, and for the pain and function subscales (0–50) 8 and 12. For pain on gripping (0–10), the SDC_95%_ was 4, and 6.4 kg for pain-free grip strength and 8.4 kg for maximum grip strength. The MIC for PRTEE total score was 9 points, 11 for pain and 4 for function. The MIC was 3.5 for pain on gripping, and 6.5 kg and 1 kg for pain-free and maximum grip strength. Construct validity was confirmed for PRTEE, pain-free grip strength, and pain on gripping. PRTEE pain and pain-free grip strength were responsive.

**Conclusion:**

All measurements were reliable. PRTEE had questionable content validity. The interpretation of MIC is challenging due to large measurement errors.

## Introduction

Tennis elbow or lateral elbow tendinopathy is a condition with pain located on or around the lateral epicondyle of the elbow. It leads to disability with high costs due to absence from work, productivity loss, and healthcare use.^1,2^ Prevalence and incidence are reported to be 1.3% yearly and 3.4 per 1000, respectively.^3,4^

The Patient-Rated Tennis Elbow Evaluation (PRTEE) is a recommended condition-specific outcome measure to evaluate pain and disability for patients with tennis elbow.^5–8^ PRTEE is translated into 13 different languages and widely used.^9^ A cross-cultural adapted version and psychometric testing are recommended in order to confirm that the version applied is an adequate reflection of the original version.^10,11^ A recently developed core-outcome set for lateral elbow tendinopathy comprises the PRTEE to measure disability.^6^ However, the PRTEE was developed without patient involvement, and little is described about its content validity and comprehensiveness.^8^

There is consistent evidence of grip strength deficits in patients with tennis elbow.^12^ Grip strength and pain on gripping are used as outcome measures and as diagnostic criteria for tennis elbow in daily clinical practice and in clinical trials.^6,13,14^ Clinical practical guidelines and a core outcome set for lateral elbow tendinopathy recommend the use of pain-free grip strength to measure physical impairment and pain.^6,7^ Despite this recommendation, only one published study with 23 participants has investigated reliability, and another study with 54 participants investigated the Minimal Important Change (MIC).^15,16^ Examining the measurement properties of pain-free grip strength and pain on gripping is highlighted as a priority for interpreting previous and future research.^17^ The maximum grip strength is tested across different musculoskeletal and neurologic conditions, but the measurement properties in tennis elbow are little investigated.^18^ To our knowledge, no measurement data exists on pain on gripping.

The study aimed to cross-cultural adapt the Norwegian version of the PRTEE and to evaluate reliability, validity, responsiveness and MIC of the PRTEE, pain-free grip strength, maximum grip strength, and pain on gripping.

## Methods

The psychometric evaluation of the Norwegian PRTEE questionnaire, pain-free grip strength, maximum grip strength, and pain on gripping were conducted with a prospective design and in accordance with the COSMIN checklist.^19^ Patients filled out the PRTEE, performed the pain-free grip strength, maximum grip strength, and reported pain on gripping at three different time points: baseline, after one week, and at the three-month follow-up. The study was approved by the Regional committees for medical and healthcare research ethics (REK109547) and registered in ClinicalTrials.gov (NCT04803825). The funders played no role in the design, conduct, or reporting of this study.

### Translation and cross-cultural adaption of the PRTEE

The PRTEE questionnaire was translated from English into Norwegian following published guidelines in December 2020.^10^ Two translators with Norwegian as their mother tongue translated the questionnaire into Norwegian. Two of the Authors (H.S. and M.B.J.) met to synthesize the translated version, and then two translators with English as their mother tongue translated the synthesized version back into English. A pre-final version was agreed on after a meeting with the translators and authors. This version was tested on 10 patients who were asked to report any difficulties in responding to the questionnaire. No amendments were made from the pre-final to the final version.

### Participants

The study included eligible patients referred to the outpatient clinic at the Department of Physical Medicine and Rehabilitation at Oslo University Hospital with tennis elbow.

The inclusion criteria were:

–  ≥18 years old
–  Two out of five clinical provocation tests had to be provocative of the symptoms on the lateral side of the elbow.^13,20^ a) Pain on palpation, b) Pain on resisted wrist extension (Cozen test), (c) Pain during power grip, (d) Pain on resisted third finger extension (Maudsley test), (e) Pain on passive elbow extension combined with wrist palmar flexion (Mills test).

The exclusion criteria were:

–  Insufficient language skills to participate.

All participants provided written informed consent before inclusion.

We aimed to include 100 patients for the validity, responsiveness, and MIC as indicated by the COSMIN study checklist as “very good”.^19^ For reliability, we aimed for at least 50 patients, which is considered adequate.^21^ Sixty of the included patients in the current study took part in a randomized controlled feasibility trial.^22^ 40 were included from the usual care treatment at the department. The usual care treatment consisted of standardized information, advice, and customized exercises.

### Outcome measures

At baseline, patients filled out a questionnaire asking about sociodemographic variables such as age, sex, mother tongue, education, work status, work type, duration of pain, and previous treatments.

#### The patient-rated tennis elbow evaluation

The PRTEE is a 15-item condition-specific Patient-Reported Outcome Measure (PROM) assessing elbow pain and disability related to tennis elbow. It consists of two subscales: (1) Pain and (2) Function. The pain subscale consists of five items rated from 0 (no pain) to 10 (worst imaginable). The function subscale consists of 6 items regarding specific activities and 4 items about usual activities, rated from 0 (no difficulty) to 10 (unable to do). A total score where pain and function are weighted equally can be computed on a scale of 0–100, where higher scores indicate more pain and disability.^5^

#### Pain-free grip strength, maximum grip strength, and pain on gripping

The pain-free grip strength and maximum grip strength are performance-based outcome measures. Various test positions are described in the literature. In this study, we tested in a standing position with a fully extended elbow in a neutral pronation/supination.^16,23^ For this study, a calibrated digital hand dynamometer (Jamar plus+) was used, and strength was registered in kg. Patients were first tested on the asymptomatic side and then on the symptomatic side. The physiotherapist used standardized instructions during the test (and was not coaching or motivating the patient in any way). Neither the physiotherapist nor the patient was able to read the dynamometer during the tests.

##### Pain-free grip strength

The pain-free grip strength was tested before the maximum grip strength. The patient holds a hand grip dynamometer, squeezes gradually, and is instructed to stop squeezing at the onset of pain; the pain-free grip strength value is registered. The measure is repeated three times with a 20-s rest interval between each measure. The total score is calculated as the mean of the three measures.

##### Maximum grip strength and pain on gripping

The patient was instructed to grip as hard as possible, the maximum grip strength was registered in kg, and pain in the elbow was verbally reported on a numeric rating scale from 0–10 (0: no pain and 10: worst imaginable pain).

### Comparator instruments

#### Quick-DASH

The Quick-Disabilities of the Arm, Shoulder, and Hand (Quick-DASH) is a region-specific, 11-item PROM, measuring physical function and symptoms related to upper-limb musculoskeletal disorders.^24–26^ Each item has five response options, from 0 (no difficulties/symptoms) to 4 (impossible/extreme symptoms). A total score from 0–100 is calculated, and a higher score indicates more disability.

#### The 5-level EuroQol-5D

The 5-Level EuroQol-5D (EQ-5D-5L) is a five-dimensional PROM measuring health-related quality of life.^27^ The dimensions are mobility, self-care, usual activities, pain/discomfort, and anxiety/depression. Each dimension is measured in five levels from 1 (no problems) to 5 (extreme problems). A total index^28^ is calculated with values that range from 1 for the best possible health state to −0.59 for the worst possible health state.

#### Global rating of change

The global rating of change (GROC) is designed to quantify a patient's improvement or worsening over time. The scale asks the patient to assess his or her current status compared to a previous time point and then calculate the difference. A GROC scale is independent from different dimensions, and the patient decides what's important.^29^ This study used a 13-point rating scale (+6: completely recovered, 0: Unchanged, −6: maximum worsening). No specific dimension of complaint (e.g., pain or function) was defined in the question. However, the question was limited to the affected elbow.

#### Anchor for responsiveness and minimal important change

At follow-up, participants were asked if they had improved since baseline (yes/no); if improved, they answered if the change was large enough to be important (yes/no). Participants who answered that their change was important were included in the improved group. Participants reporting not improved or not importantly improved were included in the unchanged group. If there was a deterioration of more than −1 on the GROC scale, the participant was excluded. The anchor was validated through correlations to GROC, PRTEE, pain-free grip strength, maximum grip strength, and pain on gripping, and a cut-off for >.30 was set as valid.^30^

#### Handling of missing values

Missing items in PRTEE were replaced by the mean score of the subscale as described in the user manual.^5,31^ The Quick-DASH allows one missing item, the score is divided by the number of responses (11 or 10) and then multiplied by 25.^25^ If a participant filled in two responses, the highest score (e.g., more disability/pain) was registered for all time points (baseline, retest and follow-up). For participants who did not respond to the question about an important change, the GROC scale was used as an anchor (+2 to +6: importantly improved and −1 to +1: unchanged).

### Statistical analysis

Statistical analyses were executed using IBM SPSS Statistics Windows, Version 29.0 Armonk, NY: IBM Corp. Mean and Standard Deviation (SD), median and Interquartile Range (IQR), and frequency (%) were reported according to the scale of the data. Analysis and terminology are reported in accordance with the COSMIN study design checklist.^19^

#### Reliability

At the re-test, participants were asked if their condition had changed since the last visit (yes/no). Only unchanged participants who were tested within two weeks from baseline were included in the analysis. A linear mixed-effects model procedure based on restricted maximum likelihood calculated the mean squares. A two-way random effects model with absolute agreement was chosen for the Intraclass Correlation Coefficient ( $ICC_{2.1}$ ). ICC values less than 0.5 were interpreted as poor reliability, 0.5 to 0.75 moderate reliability, 0.75 to 0.90 good reliability, and greater than 0.90 as excellent reliability.^32^ Absolute agreement was chosen to estimate both the Standard Error of Measurement (SEM) and the Smallest Detectable Change 95% (SDC_95%_). Bland and Altman plots were created to visually present measurement error.^33^ Cronbach´s alpha was calculated for each PRTEE subscale and the total score. Cronbach's alpha between 0.70 and 0.95 is considered good.^21^

#### Validity

Content validity of the Norwegian PRTEE was evaluated quantitatively with a questionnaire. Each question of the PRTEE was rated from 1 to 4 (1: “not relevant” to 4: “very relevant”). The item content validity index was calculated as $\frac{Number\;of\;participants\;responding\;3\;or\;4}{Total\;respondents}$ and a score >.78 was considered as excellent content validity.^34,35^ To assess the comprehensiveness, participants were asked to indicate if they felt there were any missing questions in the questionnaire.

Construct validity was evaluated through a priori hypotheses to test convergent validity. The Norwegian PRTEE, pain-free grip strength, maximum grip strength, pain on gripping, Quick-DASH, and EQ-5D-5L were correlated. The direction and magnitude of correlations were formulated, as well as the rationale for the hypotheses. A correlation of >0.5 was interpreted as measuring similar constructs, and 0.3 to 0.5 related but dissimilar constructs.^36^ A rate of ≥75% confirmed hypotheses for each measurement was considered as a confirmation of the construct validity.^21^

#### Responsiveness

Responsiveness was analyzed with a criterion and a construct approach. For the criterion approach, the anchor was used as a “gold standard,” and the Area Under the receiver operating characteristics Curve (AUC) was estimated. An AUC ≥ .70 was considered adequate.^21^ The construct approach was evaluated with a priori hypotheses about the change scores, effect size, and the standardized response mean. Change scores of PRTEE, pain-free grip strength, maximum grip strength, pain on gripping, Quick-DASH, and GROC were correlated. The direction and magnitude of correlations were formulated, as well as the rationale for the hypotheses. A correlation of >0.5 was interpreted as measuring similar constructs, and 0.3 to 0.5 related but dissimilar constructs.^36^ A rate of ≥75% confirmed hypotheses for each measurement was considered as a confirmation of the responsiveness.^21^ An effect size and standardized response mean ≥0.8 was considered a large effect size.^37^

#### Minimal important change

The MIC was calculated with an anchor-based method; it was estimated as the cut-off point on the Receiver Operating Characteristic (ROC) curve with the least misclassification between improved and unchanged patients.^30^

To evaluate the relationship between the MIC and the measurement error, Guyatt's responsiveness ratio was calculated as $\frac{MIC}{\sqrt{2xMSE}}$ .^38^ A value of >1.96 shows that the MIC lies outside the limits of agreement.^21^

## Results

100 participants were included between August 2021 and September 2023. Participants were middle-aged females (68%) with a mean age of 48 years. They were mainly in full or part-time work (57%), university-educated (72%), worked on a computer (65%), and 93% had symptoms for more than three months (Table 1). 86 participants took part in the test­–retest, 63 (73.3%) of them were unchanged since baseline. Five were excluded due to a long time between test and retest (more than two weeks). Median days from baseline to retest among the final test–retest sample (*n* = 58) were 7 (IQR 7 - 7). Five participants did not meet at the three-month follow-up and were excluded from the follow-up analysis.

**Table 1.** Demographics.

Characteristics	All (*n* = 100)
Female gender, *n*	68
Age, mean (SD)	48 (9.9)
BMI, mean (SD)	25.9 (4)
Norwegian as first language, *n*	72
Work, *n*	
Full-time	47
Part-time	10
On sick leave	35
Other social welfare benefits	8
Type of work,^a^ *n*	
Computer	65
Sitting still	63
Repeating movements	37
Large amount of lifting	27
Large amount of walking	20
Education, *n*	
Primary school	5
College	23
University	72
Symptom duration, *n*	
1–3 months	7
4–12 months	52
>12 months	41
Previous treatment, *n*	66
Bilateral elbow pain, *n*	29
Pain in dominant arm/hand, *n*	86
Number of pain sites: 0–10, median (IQR)	2 (2–4)
Ipsilateral shoulder and elbow pain, *n*	43
Patient-rated tennis elbow evaluation	
Pain (0–50), mean (SD)	24.5 (9)
Function (0–50), mean (SD)	20.3 (11)
Total (0–100), mean (SD)	44.7 (18.7)
Pain-free grip strength (kg)	
All, mean (SD)	20.9 (11)
Women, mean (SD)	19.7 (8.6)
Men, mean (SD)	23.4 (14.6)
Maximum grip strength (kg), painful side	
All, mean (SD)	29.7 (12.9)
Women, mean (SD)	25.9 (9.3)
Men, mean (SD)	38 (15.6)
Maximum grip strength (kg), unaffected side	
All (*n* = 71), mean (SD)	36.4 (11.7)
Women (*n* = 52), mean (SD)	30.9 (6.1)
Men (*n* = 19), mean (SD)	51.5 (10.1)
Pain on gripping (0–10), mean (SD)	4.8 (3.2)
EQ-5D-5L Index (−0.59–1), mean (SD)	0.58 (0.2)
EQ visual analogue scale: 0–100, mean (SD)	61 (19.7)
Quick-DASH (0–100), mean (SD)	42.5 (19.3)

SD: Standard deviation; IQR: Inter quartile range; BMI: Body mass index; EQ-5D-5L: EuroQol – 5 Dimension- 5 levels; Quick DASH: Quick Disability of the Arm; ^a^Multiple responses allowed.

### Reliability

**Figure 1.**
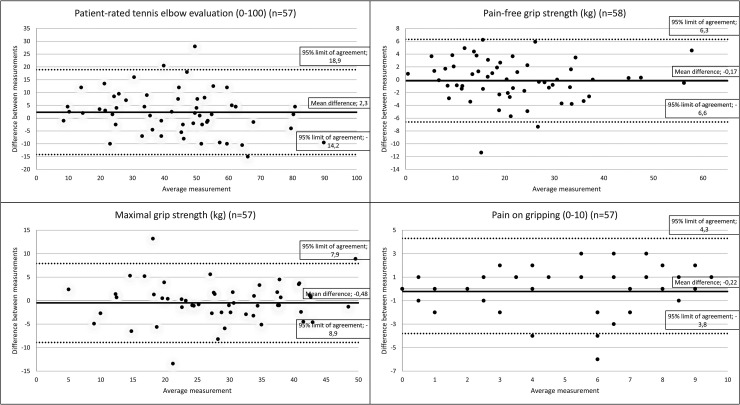
Bland and Altman plot. Illustrating the mean difference and the 95% limits of agreement for the patient-rated tennis elbow evaluation, pain-free grip strength, maximum grip strength and pain on gripping.

All outcomes achieved good to excellent reliability (Table 2). The Bland and Altman plots showed wide 95% confidence intervals for all measurements, reflecting a large measurement error. For example, the PRTEE questionnaire 95% limits were −14.2 and 18.9. Limits of Agreement are shown in the Bland Altman plot (Figure 1).

**Table 2.** Reliability (ICC, SEM, SDC_95%_), internal consistency (cronbach's alpha)).

	Score, mean (SD)			ICC (95% CI)	SEM	SDC_95%_	LoA	Cronbach´s alpha (95% CI) (*n* = 100)
	Baseline	Retest	Difference					
PRTEE pain, 0–50 (*n* = 58)	24.8 (8.9)	23.4 (9.8)	1.4 (4.1)	.90 (.82–.94)	3	8.3	−6.6 to 9.4	.86 (.81–.90)
PRTEE function, 0–50 (*n* = 57)	20.3 (11)	19.3 (11.6)	1 (5.8)	.87 (.78–.92)	4.2	11.6	−10.4 to 12.5	.93 (.90–.95)
PRTEE total, 0–100 (*n* = 57)	44.9 (18.5)	42.6 (20.3)	2.3 (8.4)	.90 (.83–.94)	6.1	17	−14.2 to 18.9	.94 (.92–.96)
Pain-free grip strength^a^, kg (*n* = 58)	21.9 (12.1)	22 (12.4)	−0.17 (3.3)	.96 (.94–.98)	2.3	6.4	−6.6 to 6.3	—
Maximum grip strength, kg (*n* = 57)	30.6 (13.1)	31.1 (13.4)	−0.48 (4.3)	.95 (.91–.97)	3	8.4	−8.9 to 7.9	—
Pain on gripping, 0–10 (*n* = 57)	4.9 (3.2)	5.2 (3.5)	−0.22 (2.1)	.81 (.70–.89)	1.5	4	−3.8 to 4.3	—

SD: standard deviation; CI: confidence interval; ICC: intraclass correlation coefficient; SEM: standard error of the measurement; SDC_95%_: smallest detectable change 95%; LoA: limits of agreement; PRTEE: Patient-Rated Tennis Elbow Evaluation; ^a^mean of three measures.

#### Internal consistency

Cronbach’s alpha for PRTEE subscales and total score were acceptable (range 0.86–0.94) (Table 2).

### Content validity

A total of 61 participants responded to the questionnaire evaluating the content validity of the PRTEE. The item content validity index ranged from .52 to .92. Eight items (4, 6, 8, 9, 10, 11, 12 and 13) were below the criteria for excellent content validity (>.78). A table with responses per item is provided in Supplemental Appendix 1. 24 (39%) participants reported that there were relevant questions missing. The most frequently reported theme was night pain/problems sleeping and using a smartphone. A full list of questions reported as relevant is provided in Supplemental Appendix 2.

### Construct validity

PRTEE, pain-free grip strength, and pain on gripping were above the 75% cut-off for confirmed hypotheses. For maximum grip strength, 50% (2/4) of the hypotheses were confirmed. A full list of hypotheses and correlations is presented in Table 3.

**Table 3.** Predetermined hypotheses to evaluate the construct validity.

Hypothesis and rationale	Calculated Value (*P*-value)	Hypothesis confirmed
Construct validity:		
Very strong positive correlation (>0.7) between PRTEE total and Quick-DASH (*n* = 99). *Rationale:* Measuring arm pain and disability. 11/13 studies correlated very strong PRTEE total and DASH.^39–51^	rho. .85 (<.001)	Yes
Strong negative correlation (−0.5–−0.7) between PRTEE total and EQ-5D-5L Index score (*n* = 99). *Rationale:* Health related quality of life is strongly related to pain and disability. One previous study correlated strong PRTEE total and EQ-5D-5L.^47^	rho. −.74 (<.001)	No
Moderate negative correlation (−0.3–−0.5) between PRTEE total and pain-free grip strength (*n* = 100). *Rationale:* PRTEE measures a different construct, but is related to pain-free grip strength. 3/3 studies correlated moderate PRTEE total and pain-free grip strength.^52–54^	rho. −.50 (<.001)	Yes
Strong positive correlation (0.5–0.7) between PRTEE pain and pain on gripping (*n* = 100). *Rationale:* Pain on gripping is measuring the same construct as PRTEE pain. 3/3 studies correlated strong VAS pain and PRTEE pain.^40,42,53^	rho. .53 (<.001)	Yes
Moderate negative correlation (−0.3–−0.5) between pain-free grip strength and pain on gripping (*n* = 100). *Rationale:* Performance in pain-free grip strength is a different construct, but related to pain.	rho. −.46 (.002)	Yes
Strong positive correlation (0.5–0.7) between pain-free grip strength and maximum grip strength (*n* = 100). *Rationale:* Both measures gripping performance.	rho. .70 (<.001)	Yes
Moderate negative correlation (−0.3–−0.5) between pain-free grip strength and Quick-DASH (*n* = 99). *Rationale:* Performance in pain-free grip strength is a different construct, but related to arm pain and disability.	rho. −.41 (<.001)	Yes
Moderate positive correlation (0.3–0.5) between pain-free grip strength and EQ-5D-5L Index score (*n* = 99). *Rationale:* Performance in pain-free grip strength is a different construct, but related to arm pain and disability, and health related quality of life.	rho. .32 (.001)	Yes
Moderate negative correlation (0.3–0.5) between maximum grip strength and *Quick DASH* (*n* = 99) *Rationale:* Previous research found Grip strength to correlate with Quick DASH.^55^	rho. −.40 (<.001)	Yes
Moderate negative correlation (0.3–0.5) between maximum grip strength and pain on Gripping (*n* = 100). *Rationale:* Pain reduces grip strength.	rho. −.26 (.009)	No
Moderate negative correlation (0.3–0.5) between maximum grip strength and *EQ-5D-5L (n* *=* *99). Rationale:* Grip strength is related to health related quality of life.^56^	rho. 27 (.007)	No
Moderate negative correlation (−0.3–−0.5) between pain on gripping and Quick-DASH (*n* = 99). *Rationale:* Pain on gripping is a different construct, but related to Quick-DASH.	rho. .34 (<.001)	Yes
Moderate negative correlation (−0.3–−0.5) between pain on gripping and EQ-5D-5L Index score (*n* = 99) *Rationale:* Pain on gripping is a different construct, but related to health related quality of life.	rho. 40 (<.001)	Yes

PRTEE: Patient-Rated Tennis Elbow Evaluation; Quick-DASH: Quick Disability of the Arm and Hand; EQ-5D-5L: EuroQol-5 Dimension—5 levels, Visual analogue scale; GROC: global rating of change; ES: effect size; SRM: standardized response mean.

### Responsiveness

#### Validation of the anchor

The correlation between the anchor and GROC, change scores of PRTEE total score, pain-free grip strength, maximum grip strength, and pain on gripping were .74, .47, .33, .18, and .28, respectively. Sixty participants considered their changes as important. Four participants did not respond to the question about an important improvement but were included in the improved group based on using the GROC scale as an anchor (Table 4). Five participants rated less than −1 on the GROC scale, and they were considered deteriorated and excluded from the analyses (Table 4).

**Table 4.** Distribution of participants in the improved and unchanged group by the anchor question and global rating of change.

GROC	Important change, *n*			Total, *n*
	Yes	No	Missing	
6	7	0	1	8
5	17	0	1	18
4	17	1	0	18
3	10	0	2	12
2	4	1	0	5
1	5	10	1	16
0	0	11	0	11
−1	0	2	0	2
−2	0	1	0	1
−3	0	1	0	1
−4	0	3	0	3
−5	0	0	0	0
−6	0	0	0	0
Total	60	30	5	95

Participants < −1 on the GROC scale were excluded from the analyses. Grayed out cells: included into the improved group. GROC: Global Rating Of Change.

#### Criterion responsiveness

**Figure 2.**
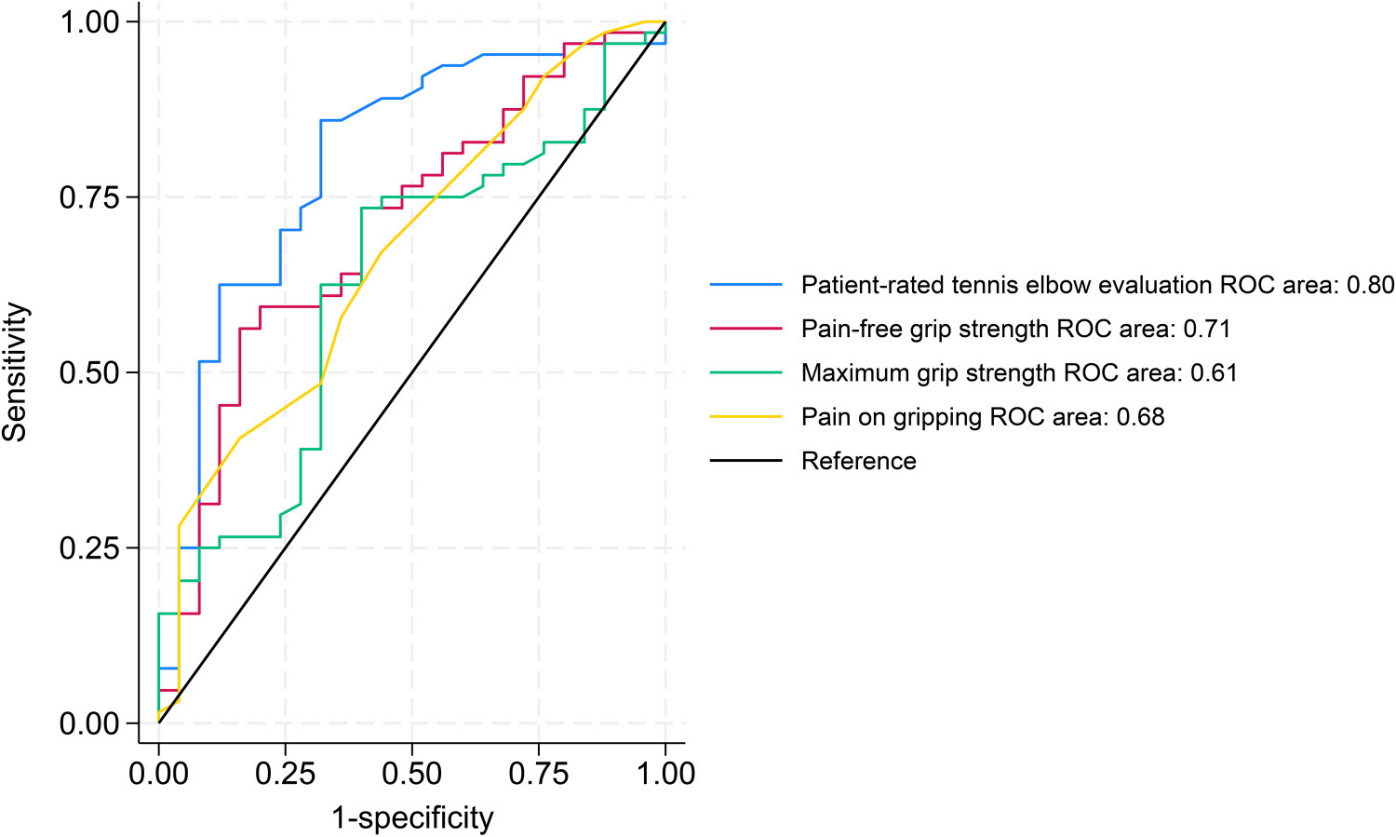
The receiver operating characteristic curves of the Patient-Rated Tennis Elbow Evaluation, pain-free grip strength maximal grip strength and pain on gripping at three month follow-up.

The AUC was above the 0.7 cut-off for PRTEE pain, PRTEE function, PRTEE total, and pain-free grip strength. Maximum grip strength and pain on gripping were below the 0.7 cut-off for AUC (Table 5). The ROC curves are presented in Figure 2.

**Table 5.** Baseline, follow-up, change scores, effect size (ES) and standardized response mean (SRM).

	PRTEE Pain (0–50)	PRTEE function (0–50)	PRTEE total (0–100)	Pain-free grip strength (kg)	Maximum grip strength (kg)	Pain on gripping (0–10)
Baseline score, mean (SD)						
Improved (*n* = 64)	23.2 (9.5)	19.5 (10.6)	42.7 (18.9)	21.2 (11.9)	30.1 (12.7)	4.8 (3.2)
Unchanged (*n* = 26)	27.2 (7.2)	23.6 (11.5)	50.8 (17)	20.8 (9.4)	28.8 (12)	4.3 (3.3)
Follow-up score, mean (SD)						
Improved (*n* = 64)	10.9 (9.7)	6.7 (8.2)	17.6 (17.1)	30.8 (11.3)	34.9 (11.9)	2.2 (2.8)
Unchanged (*n* = 26)	24.2 (8.1)	16.8 (11.5)	40.9 (18.4)	24.1 (10.7)	30.2 (13.7)	3.7 (3.4)
Change score, mean (SD)						
Improved (*n* = 64)	12.3 (9.1)	12.8 (8.7)	25.1 (16.3)	9.6 (10.5)	4.8 (8.5)	2.6 (3.1)
Unchanged (*n* = 26)	3.1 (4.9)	6.4 (9)	9.3 (12.6)	3.2 (9)	1.4 (5.4)	0.6 (3.2)
Effect size						
Improved (*n* = 64)	1.30	1.20	1.33	0.80	0.38	0.81
Unchanged (*n* = 26)	0.43	0.56	0.55	0.34	0.1	0.18
Standardized response mean						
Improved (*n* = 64)	1.35	1.47	1.54	0.91	0.56	0.84
Unchanged (*n* = 26)	0.63	0.71	0.74	0.36	0.26	0.19
AUC (95% CI)	0.81 (0.73 to 0.90)	0.76 (0.64 to 0.88)	0.80 (0.70 to 0.91)	0.71 (0.59 to 0.83)	0.61 (0.49 to 0.74)	0.68 (0.56 to 0.80)
MIC (sens./spec.)	10.5 (0.59 / 0.96)	3.5 (0.92 / 0.56)	9 (0.86 / 0.68)	6.5 (0.56 / 0.85)	1 (0.73 / 0.58)	3.5 (0.41 / 0.85)
Responsiveness ratio	2.57	0.60	1.07	1.98	0.23	1.70

SD: standard deviation; CI: confidence interval; PRTEE: Patient-Rated Tennis Elbow Evaluation; AUC: area under the receiver operating characteristic curve; MIC: minimal important change, Responsiveness ratio calculated as $\frac{MIC}{\sqrt{2xMSE}}$

#### Construct responsiveness

The PRTEE questionnaire, pain-free grip strength, and pain on gripping were above the 75% cut-off for confirmed hypotheses. Only 25% of the hypotheses for maximum grip strength were confirmed. A full list of hypotheses and correlations is presented in Table 6.

**Table 6.** Predetermined hypotheses to evaluate the construct responsiveness.

Hypothesis and rationale	Calculated Value (*P*-value)	Hypothesis confirmed
*Responsiveness:*		
Very strong positive correlation (0.7–0.9) of change scores between PRTEE total and Quick-DASH (*n* = 93). *Rationale:* Measuring change in same construct.	rho. .78 (<.001)	Yes
Strong positive correlation (0.5–0.7) of change scores between PRTEE pain and pain on gripping (*n* = 94). *Rationale:* Measuring change in pain related to elbow.	rho. .53 (<.001)	Yes
Strong positive correlation (0.5–0.7) of change scores between maximum grip strength and pain-free grip strength (*n* = 90) *Rationale:* Measuring change in gripping.	rho. 59 (<.001)	Yes
Moderate correlation (0.3–0.5) of change scores between PRTEE total and pain-free grip strength. *Rationale:* (*n* = 94)*:* Change in pain-free grip strength is related to change in elbow pain and disability, but differ in construct.	rho. .42 (<.001)	Yes
Moderat positive correlation (0.3–0.5) of change scores between maximum grip strength and *Quick-DASH (n* **=** 90).^55^	rho. .23 (.028)	No
Moderate correlation (0.3–0.5) of change scores between pain-free grip strength and pain on gripping (*n* = 94). *Rationale:* Change in pain-free grip strength is related to elbow pain on gripping.	rho. .45 (<.001)	Yes
Strong positive correlation (0.5–0.7) between PRTEE total change and GROC (*n* = 94). *Rationale:* Both reflecting change of the elbow.	rho. .63 (<.001)	Yes
Strong negative correlation (0.5–0.7) between pain-free grip strength change and GROC (*n* = 95). *Rationale:* Both reflecting change of the elbow.	rho. .29 (.004)	No
Strong negative correlation (0.5–0.7) between maximum grip strength change and GROC (*n* = 94). *Rationale:* Both reflecting change of the elbow.	rho .092 (.389)	No
Strong positive correlation (0.5–0.7) between pain on gripping change and GROC (*n* = 94). *Rationale:* Both reflecting change of the elbow.	rho. .40 (<.001)	No
ES and SRM in PRTEE total is >0.8 for the improved group.	ES: 1.3 SRM: 1.5	Yes
ES and SRM in pain-free grip strength is >0.8 for the improved group.	ES: 0.8 SRM: 0.9	Yes
ES and SRM in maximum grip strength is >0.8 for the improved group.	ES: 0.4 SRM: 0.6	No
ES and SRM in pain on gripping is >0.8 for the improved group	ES: 0.8 SRM: 0.8	Yes

PRTEE: Patient-Rated Tennis Elbow Evaluation; Quick-DASH: Quick Disability of the Arm and Hand; EQ-5D-5L: EuroQol-5 Dimension—5 levels; VAS: Visual analogue scale; GROC: Global Rating of Change; ES: Effect size; SRM: Standardized response mean.

### Minimal important change

The ROC curves show that the MIC for PRTEE pain subscale and pain-free grip strength were above the 1.96 cut-off for Guyatt's responsiveness ratio. Minimal important changes for each outcome measure are shown in Table 5. The MIC distributions are shown in Supplemental Appendix 3 and show the misclassification of the MIC.

## Discussion

This study successfully translated and cross-cultural adapted the English version of the patient-rated tennis elbow evaluation into Norwegian. The sample size was in accordance with the COSMIN checklist. To the best of our knowledge, this is the first study in patients with tennis elbow to investigate the content validity of the PRTEE questionnaire, the measurement properties of pain on gripping, and the absolute test–retest reliability of maximum grip strength. Also, it is the largest study testing the measurement properties of the pain-free grip strength. All measures were reliable, showing an ICC >0.8. However, the measurement errors were quite high. Hypothesis testing showed that all measures except the maximum grip strength had adequate construct validity and construct responsiveness. Only the PRTEE pain subscale and pain-free grip strength were above the responsiveness ratio cut-off. Hence, the MIC calculated for these two outcomes can be considered a true change.

### The patient-rated tennis elbow evaluation

The questionnaire showed acceptable values for reliability. However, compared to a systematic review and meta-analysis on PRTEE, the results are below the pooled ICC and Cronbach’s Alpha of 0.96, and above the pooled SEM and SDC_95%_ values of 3.5 and 9.^9^ The ROC curves (Figure 2) demonstrate that the PRTEE is the most sensitive outcome measure for detecting change. This supports the recommendation of PRTEE as the primary outcome for tennis elbow and highlights the importance of using patient-reported outcome measures.^6^

The MIC estimated in this study confirms two previous studies calculating the MIC to be between 7 and 11.^43,57^ However, the high SDC_95%_ of 17 points for the PRTEE total in this study makes the interpretation of MIC (9 points) impractical for research and clinical practice, since it is impossible to discriminate between a random error or a meaningful change.^58^ The MIC distribution curves (Supplemental Appendix 3) show the closeness between the improved and unchanged groups and the overlap above the MIC for the unchanged group. The proximity between the groups makes it more likely that the MIC is small and falls within the boundaries of measurement error.^59^ A method to increase the MIC value is to set the cut-off for change higher.^57^ However, this is not recommended as the value and then no longer represents the minimal change.^58^

Content validity is considered to be the most important measurement property, yet PRTEE was developed without patient involvement.^60^ More than half of the items were found below the cut-off for excellent content validity in this study, and confirms the comment in the core outcome set that “*some items in the PRTEE-function may not represent their experience.*”^6^ We found that the most reported missing theme was night pain/problems sleeping. This confirms what Bateman et al.^61^ reported in the recent qualitative study on tennis elbow and is not surprising, as musculoskeletal pain is associated with increased risk of sleep problems.^62^ The second and third most reported theme was using a smartphone and a computer. The smartphone was not introduced at the time of the PRTEE development in 2005. However, with the increased use of smartphones and computers in private life and work, these themes should be included in the questionnaire. The findings in this study suggest that the PRTEE is not an adequate reflection of the tennis elbow pain and disability construct for the current study population.

However, the methods chosen and the study population likely influence the results on content validity. Different cut-offs (0.70–0.80) for the content validity have been suggested, and 0.78 is considered evidence of good content validity.^34,35^ But, the cut-offs are described for smaller sample sizes (0–9 participants), and the transferability of cut-offs to larger samples might not be precise. The 0.78 cut-off can be considered arbitrary and too conservative. E.g., lowering the cut-off to 0.70 leads to three more items being relevant (Supplemental Appendix 1). The study population predominantly consisted of individuals with mild to moderate symptoms. Thus, items of lower difficulty were perceived as less relevant. It is plausible that many participants who selected “somewhat relevant” might have rated the items as more relevant if they had experienced a more severe case of tennis elbow. Nuances in the responses are lost due to dichotomization, and including “somewhat relevant” as relevant leads to only two items being below the 0.78 cut-off (Supplemental Appendix 1). The generalizability of these findings is therefore questionable, indicating a need for additional research on content validity. However, item 6: Turning a doorknob/key and item 10: Pull up pants are still not relevant in any variant of post-hoc analyses (Supplemental Appendix 1).

### Pain-free grip strength

Pain-free grip strength showed good results in all the properties tested, with the MIC outside the limits of agreement. The current study found that pain-free grip strength had slightly better reliability than what Hill et al.^15^ found in their sample of 23 individuals with an ICC of 0.94 and a SDC_95%_ of 9.4 kg. The Bland-Altman plot for pain-free grip strength shows a more positive difference in the lower scores and a negative difference in the higher scores. This could be explained by experiences from the baseline testing. Patients with higher scores allowed themselves to press harder at retest and the opposite at lower scores. Pain-free grip strength correlated moderately with PRTEE, confirming previous research.^52–54^ The correlation indicates a related construct, confirming the recommendation of using PRTEE to measure physical impairment and pain.^6^ The results from this study confirm the clinically important change of 7 kg for pain-free grip strength that Stratford and Levy^16^ found in their sample of 54 patients in 1994. The estimated MIC had low sensitivity, indicating a high degree of false negatives, which is visualized by the overlap of improved patients below the MIC on the distribution curve. Hence, when using this MIC value, one should be aware of the somewhat lower ability to discriminate importantly changed patients. But at 6.5 kg, there is high specificity, and one can be confident that there is a low chance of a false positive above this value.

### Maximum grip strength

Maximum grip strength was reliable, and ICC was comparable to pooled results from a recent systematic review on grip strength for upper extremity conditions of 0.96.^18^ The mean maximum grip strength increased from baseline to follow-up. The mean maximum grip strength ratio for improved patients at follow-up was 0.97 (based on the mean baseline score of the non-affected arm), which is as good as reported in healthy volunteers.^55^ However, maximum grip strength was not found to be responsive in this study. The effect size, standardized response mean, and AUC were all below the cut-off values. The correlation of the change score and the anchor was low and confirms the previously reported correlation to the global rating of change (0.23).^16^ The improvement was small, revealing that participants performed well at baseline despite their pain. This could also explain why it did not correlate in the testing of construct validity. The measurement properties of the grip strength ratio were not tested in this study and could have given another result.

### Pain on gripping

Pain on gripping correlated strongly with the PRTEE pain subscale and 80% of the pre-defined hypotheses were confirmed. However, pain on gripping was not responsive in this study and cannot be recommended for measuring change. The core outcome set favored the pain on gripping over the PRTEE pain subscale.^6^ However, the recommendations were made before measurement data on pain on gripping existed. In the current study, the PRTEE pain subscale showed excellent responsiveness in contrast to pain on gripping. These findings challenge the core outcome set and indicate that the PRTEE pain subscale could be a recommended measure for pain.

### Limitations

We did not perform any cognitive interviews, although recommended by the COSMIN checklist. Interviews could have given valuable information about the eight items that were below the cut-off for excellent content validity. The correlation of the anchor and the change scores of the maximum grip strength and pain on gripping was below the cut-off. This weakens the validity of the results. However, PRTEE and pain-free grip strength correlated above the cut-off. Hence, indicating that the anchor itself was valid. Maximum grip strength was only measured once and performed after testing the pain-free grip strength. Investigating the mean of three could improve the reliability, but would probably not change the result of validity or responsiveness, which was the problem for maximum grip strength. As much as 43% had ipsilateral shoulder and elbow pain, which might have affected the construct validity since the construct of pain and disability for elbow and shoulder pain may overlap. Also, this could explain the strong correlation between PRTEE and Quick-DASH. However, rotator cuff pathology has been reported as a risk factor with an odds ratio as high as 4.95.^63^ Thus, shoulder pain is probably normal for many with elbow pain. The population in the current study was recruited from secondary care, with 41% reporting elbow pain for more than 12 months. The results should be interpreted in this context and the external validity assessed accordingly.

## Conclusion

All measurements had acceptable reliability. PRTEE had questionable content validity. The interpretation of MIC is challenging due to large measurement errors. The PRTEE pain subscale and Pain-free grip strength were the only measurements found to be responsive.

## Supplemental Material

sj-docx-1-sel-10.1177_17585732251344264 - Supplemental material for Measurement properties of core outcomes in patients with tennis elbow Supplemental material, sj-docx-1-sel-10.1177_17585732251344264 for Measurement properties of core outcomes in patients with tennis elbow by Håkon Sveinall, Jens Ivar Brox, Kaia B Engebretsen, Aasne Fenne Hoksrud, Cecilie Røe and Marianne Bakke Johnsen in Shoulder & Elbow
