# Supplementary material for: Measurement properties of core outcomes in patients with tennis elbow
Source: Shoulder Elbow. 2025 May 29;18(4):835–48. doi: 10.1177/17585732251344264 (PMC12122481; doi:10.1177/17585732251344264)
Supplement: sj-docx-1-sel-10.1177_17585732251344264 - Supplemental material for Measurement properties of core outcomes in patients with tennis elbow [file sj-docx-1-sel-10.1177_17585732251344264.docx]

**Appendix 1:** Responses to relevance per item of the Norwegian Patient-rated tennis elbow evaluation.

| Item | 1:  Not  Relevant | 2: Somewhat  Relevant | 3:  Quite  relevant | 4: Highly relevant | (2 - 4) / total | 2 - 4 I-CVI | (3 - 4) / total | 3 – 4 I-CVI |
| --- | --- | --- | --- | --- | --- | --- | --- | --- |
| 1: When you are at rest | 4 | 8 | 17 | 32 | 57/61 | .93 | 49/61 | .80 |
| 2: When doing a task with repeated arm movement | 0 | 5 | 11 | 45 | 61/61 | 1 | 56/61 | .92 |
| 3: When carrying a plastic bag of groceries | 2 | 5 | 15 | 39 | 59/61 | .97 | 54/61 | .89 |
| 4: When your pain was at its least | 5 | 12 | 14 | 30 | 56/61 | .92 | 44/61 | .72 |
| 5: When your pain was at its worst | 2 | 3 | 16 | 39 | 58/60 | .97 | 55/60 | .92 |
| 6: Turn a doorknob or key | 15 | 14 | 15 | 16 | 45/60 | .75 | 31/60 | .52 |
| 7: Carry a grocery bag or briefcase by the handle | 3 | 5 | 19 | 34 | 58/61 | .95 | 53/61 | .87 |
| 8: Lift a full coffee cup or glass of milk to your mouth | 11 | 12 | 11 | 25 | 48/59 | .81 | 36/59 | .61 |
| 9: Open a jar | 4 | 10 | 19 | 27 | 56/60 | .93 | 46/60 | .77 |
| 10: Pull up pants | 20 | 14 | 13 | 13 | 40/60 | .67 | 26/60 | .43 |
| 11: Wring out a washcloth or wet towel | 8 | 14 | 20 | 19 | 53/61 | .87 | 39/61 | .64 |
| 12: Personal activities (dressing, washing) | 13 | 12 | 14 | 22 | 48/61 | .79 | 36/61 | .60 |
| 13: Household work (cleaning, maintenance) | 6 | 11 | 10 | 34 | 55/61 | .90 | 44/61 | .72 |
| 14: Work (your job or everyday work) | 1 | 7 | 9 | 44 | 60/61 | .98 | 53/61 | .87 |
| 15: Recreational or sporting activities | 3 | 8 | 14 | 36 | 58/61 | .95 | 50/61 | .82 |
| PRTEE S-CVI: |  |  |  |  |  | 0.83 |  | 0.74 |
| Pain-free grip strength | 3 | 9 | 18 | 31 | 58/61 | .95 | 49/61 | .80 |

I-CVI: Item – Content Validity Index, S-CVI: Scale – Content Validity Index

**Appendix 2:** Themes and number of times reported missing from the patient-rated tennis elbow evaluation

| Themes | Number of times | Possible classification in the current PRTEE* |
| --- | --- | --- |
| Night pain/ problems sleeping | 6 |  |
| Phone or smartphone | 5 | Work or recreational |
| Computer | 3 | Work or recreational |
| Dress on the upper body | 3 | Personal activities |
| Pain after activity | 3 |  |
| Riding a bike | 2 | Recreational or sporting activities |
| Bending the arm | 1 | “Lift a full cup” |
| Sit and rest | 1 | Question 1 |
| Cary children or weight on the arm | 1 |  |
| Specific activity | 1 | Usual activities in general |
| Hang laundry | 1 | Household work |
| Drive a car | 1 | Usual activities in general |
| Traveling / be social | 1 | Recreational activities |
| Exercises, treatment or aids | 1 | Recreational or sporting activities |
| Physical work | 1 | Work |
| Write with a pen | 1 | Work or recreational |
| Specify weight of bag / suitcase | 1 | Specific activities |
| Gripping | 1 | “Wring out a washing cloth” |
| Ergonomic positions at work | 1 | Work |
| Adaptation of activities due to pain | 1 | Usual activities in general |
| Shower / shampooing | 1 | Personal activities |
| Personal care: shaving, brushing teeth, cutting nails. | 1 | Usual activities |
| Heavy lifting | 1 |  |
| Shoulder pain | 1 |  |
| Fluctuations in pain during activity | 1 |  |
| Pick something up from jacket pocket | 1 | Personal activities |

*Thems that can be considered already covered indirectly by other items in the questionnaire.
